# Device simulation of highly efficient eco-friendly CH_3_NH_3_SnI_3_ perovskite solar cell

**DOI:** 10.1038/s41598-021-82817-w

**Published:** 2021-02-04

**Authors:** Piyush K. Patel

**Affiliations:** Renewable Energy Laboratory, Department of Physics, Maulana Azad National Institute of Technology, Bhopal, M. P. India

**Keywords:** Materials science, Physics

## Abstract

Photoexcited lead-free perovskite CH_3_NH_3_SnI_3_ based solar cell device was simulated using a solar cell capacitance simulator. It was modeled to investigate its output characteristics under AM 1.5G illumination. Simulation efforts are focused on the thickness, acceptor concentration and defect density of absorber layer on photovoltaic properties of solar cell device. In addition, the impact of various metal contact work function was also investigated. The simulation results indicate that an absorber thickness of 500 nm is appropriate for a good photovoltaic cell. Oxidation of Sn^2+^ into Sn^4+^ was considered and it is found that the reduction of acceptor concentration of absorber layer significantly improves the device performance. Further, optimizing the defect density (10^14^ cm^−3^) of the perovskite absorber layer, encouraging results of the *J*_*sc*_ of 40.14 mA/cm^2^, *V*_*oc*_ of 0.93 V, *FF* of 75.78% and *PCE* of 28.39% were achieved. Finally, an anode material with a high work function is necessary to get the device's better performance. The high-power conversion efficiency opens a new avenue for attaining clean energy.

## Introduction

In the future, energy demand will be increased drastically. Nowadays, most of the energy consumption is obtained from fossil fuels, and this energy reserve will be depleted in the coming days. Since it pollutes the environment, therefore, the most challenging task is to establish a renewable energy source. The demand for renewable sources of energy is increasing due to industrialization and growing populations. For this, solar energy is an up-and-coming source because it is clean and no adverse effect on the environment^1–7^. Solar energy devices after a sustainable solution in global energy are demanded.

Perovskite solar cell (PSC) has garnered tremendous attention to the scientific community due to increased power conversion efficiency (*PCE*) day by day. Researchers started working on perovskite solar cells in 2009^8^. At that time, efficiency was relatively low (~ 3.8%). This organic–inorganic perovskite solar cell becomes a next-generation device because it showed a step increase in *PCE.* The state-of-the-art certified *PCE* of PSC is exceeding 25%^5–7,9,10^. Apart from that, these materials exhibit peculiar features such as high absorption coefficient, good charge carrier mobility, small exciton binding energy and large diffusion length of charge carriers^9,11–13^. Therefore, it is imperative to work on it. This energy can be converted into electricity using a photovoltaic effect^14^. However, it is cheap and clean energy; hence, it has dominated the Si-based solar cell in the photovoltaic market^15^. People studied pure and modified methylammonium lead halide as a perovskite material because of their good photovoltaic performance^16,17^. Nowadays, some of the reports are coming on lead-based materials. However, lead-based electronic devices have been sternly circumscribed by the European Union and other countries as well. These materials showed a high value of power conversion efficiency. However, lead is not eco-friendly, which is harmful to humans and the environment^5,18–25^.

To overcome this issue and owing to the superior optoelectronic properties lead-free perovskite CH_3_NH_3_SnI_3_ has been explored by many scientists and researchers as a photovoltaic material. This material exhibits a direct band gap of 1.3 eV, a suitable range for the absorber. Recently, several group have successfully fabricated/simulated organic–inorganic perovskites solar cells based CH_3_NH_3_SnI_3_, which yields the good *PCE*. Freshly, the studied description stated that CH_3_NH_3_SnI_3_ is as favorable as its lead-based counterpart^17,26–30^. The electron transport layer (ETL) is a crucial component of PSC. TiO_2_ is a promising candidate material because of its appropriate energy level for electron injection, high electron mobility, chemical stability, low synthesis cost and environmental friendliness^31–34^. TiO_2_ material has an appropriate band gap between diminishing the transportation of holes^35^. The hole transport layer (HTL) plays a crucial role for getting the high-power conversion efficiency in PSC. HTL needs high hole carrier mobility and should form a less defect at the HTL/absorber layer to minimize the charge carriers recombination at the interface. Recently, Yu et al. reported that Cu_2_O as an HTL showed high hole mobility, good energy level alignment with CH_3_NH_3_PbI_3_ and a longer lifetime of photo-generated charge carriers^33^. Cu_2_O is used as an HTL because of abundant availability on Earth, environmental-friendly, perfectly band alignment with CH_3_NH_3_SnI_3_ and easily synthesized materials. It decreases the barrier height of metal contact and reduces the recombination loss of minority at anode^11,36,37^. Device simulation provides a strong way to improve PSC's efficiency after the optimization of various physical parameters. Solar cell capacitance simulator (SCAPS) was utilized by many theoreticians to predict the open circuit voltage (*V*_*oc*_), short circuit current density (*J*_*sc*_), fill factor (*FF*) and *PCE* of the perovskite based solar cell^27,38,39^. Hence, the simulation of lead-free CH_3_NH_3_SnI_3_ as a photoactive material was studied using SCAPS. The impact of rectifying and ohmic contact behaviour on lead-free CH_3_NH_3_SnI_3_ based PSC was also investigated.

## Device structure and simulation

In this work, a numerical simulation of a planar heterojunction tin-based perovskite solar cell was performed using SCAPS. To obtain the performance parameters of the device like current density–voltage (*J*-*V*) curve, quantum efficiency and energy bands, Poisson Eq. (1) is solved with continuity equation of electron (2) and hole (3). These curves are used to calculate *J*_*sc*_, *V*_*oc*_, *FF* and *PCE* of the solar cell device.

1 $$\frac{d}{dx}\left(-\varepsilon (x)\frac{d\psi }{dx}\right)=q[p\left(x\right)-n\left(x\right)+{N}_{D}^{+}\left(x\right)-{N}_{A}^{-}\left(x\right)+{p}_{t}\left(x\right)-{n}_{t}(x)]$$

2 $$\frac{d{p}_{n}}{dt}={G}_{p}-\frac{{p}_{n}-{p}_{n0}}{{\tau }_{p}}+{p}_{n}{\mu }_{p}\frac{d\xi }{dx}+{\mu }_{p}\xi \frac{d{p}_{n}}{dx}+{D}_{p}\frac{{d}^{2}{p}_{n}}{d{x}^{2}}$$

3 $$\frac{d{n}_{p}}{dt}={G}_{n}-\frac{{n}_{p}-{n}_{p0}}{{\tau }_{n}}+{n}_{p}{\mu }_{n}\frac{d\xi }{dx}+{\mu }_{n}\xi \frac{d{n}_{p}}{dx}+{D}_{n}\frac{{d}^{2}{n}_{p}}{d{x}^{2}}$$

where, *G*, *τ*_*n*_, *τ*_*p*_, *D*, *q*, *Ψ*, *µ*_*n*_, *µ*_*p*_, *n* (*x*), *p* (*x*), *n*_*t*_ (*x*), *p*_*t*_ (*x*), ${N}_{A}^{-}\left(x\right)$, ${N}_{D}^{+} (x)$ and *ξ* denote the generation rate, electron life time, hole life time, diffusion coefficient, electron charge, electrostatic potential, electron mobility, hole mobility, concentration of free electrons, concentration of free holes, concentration of trapped electrons, concentration of trapped holes, ionized acceptor concentrations, ionized donor concentrations and electric field, respectively. *x* denotes the direction along the thickness^29^.

**Figure 1:**
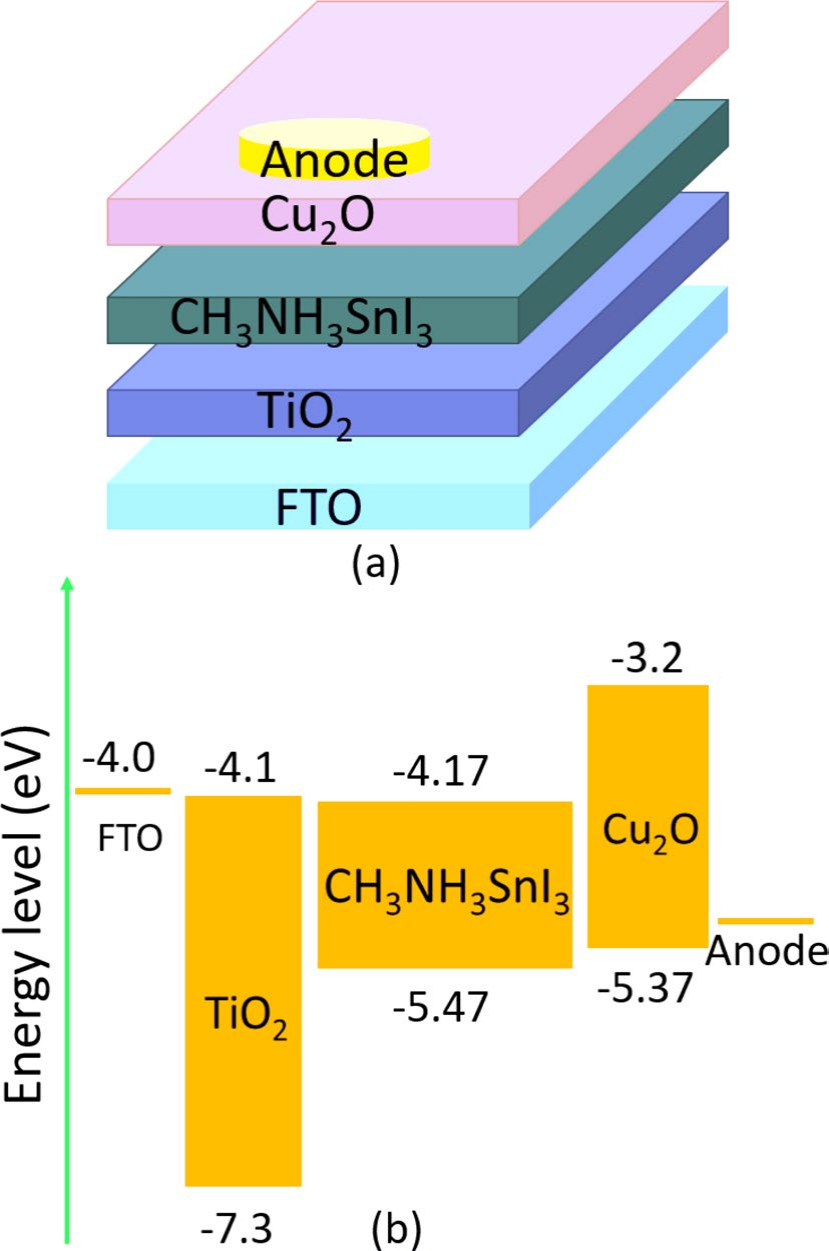
(**a**) Lead-free CH_3_NH_3_SnI_3_ solar cell structure. (**b**) Energy level diagram.

The device's structure in the simulation is transparent conduction oxide (TCO)/buffer (ETL)/interface defect layer 1/absorber/interface defect layer 2/HTL. The simulation was done under the illumination of 1000 W/m^2^, at 300 K and an air mass of AM 1.5G. The active area of the studied device is 1 cm^2^. The device's configuration is illustrated in Fig. 1a where, *p*-type Cu_2_O is used as an HTL, CH_3_NH_3_SnI_3_ is used as an absorber layer and *n*-type TiO_2_ is used as an ETL. In addition, fluorine doped tin oxide (FTO) is selected as the contact material and various materials like Ag, Cu, Au and Pt is selected as an anode. The energy level diagram of the corresponding materials utilized in the device architecture is depicted in Fig. 1b.

The values of the device and material parameters that are adopted from theories, experiments and literature are summarized in Tables 1 and 2^27–29,39^. Initially, the thickness of FTO (500 nm), TiO_2_ (120 nm) and Cu_2_O (420 nm) were optimized for high *PCE*, as mentioned in Table 1.

Herein, *χ* is the electron affinity, *E*_*g*_ is the band gap, *ε*_*r*_ is the relative permittivity, *N*_*c*_ is the density of state of the conduction band, *N*_*v*_ is the density of state of the valence band, *µ*_*n*_ is the mobility of electron, *µ*_*p*_ is the mobility of hole, *N*_*A*_ is the acceptor density, *N*_*D*_ is the donor density and *N*_*t*_ is the defect density. The thermal velocity of electron and hole are set be 10^7^ cm/s. The absorption coefficient of FTO, Cu_2_O, CH_3_NH_3_SnI_3_ and TiO_2_ were extracted from experimental results^15,40–42^. The diffusion lengths of electron and hole were set to 260 nm and 560 nm, respectively, similar to the experimentally observed value of Ma et al.^28^.

**Table 1:** Parameters for the optoelectronic simulation.

Parameters	FTO (TCO)	TiO_2_ (ETL)	CH_3_NH_3_SnI_3_ (absorber)	Cu_2_O (HTL)
Thickness (nm)	500	120	450 (variable)	420
*E*_*g*_ (eV)	3.4	3.2	1.3	2.17
*χ* (eV)	4.5	4.1	4.17	3.2
*ε*_*r*_	9.1	9.0	8.2	7.1
*N*_*c*_ (cm^−3^)	1.1 × 10^19^	2.2 × 10^18^	1 × 10^18^	2 × 10^17^
*N*_*v*_ (cm^−3^)	1.1 × 10^19^	1.8 × 10^19^	1 × 10^18^	1.1 × 10^19^
*µ*_*n*_ (cm^2^/Vs)	20	0.05	2000	200
*µ*_*p*_ (cm^2^/Vs)	10	0.05	300	80
*N*_*D*_ (cm^−3^)	1.1 × 10^19^	1 × 10^18^	0	0
*N*_*A*_ (cm^−3^)	0	0	1 × 10^14^ (variable)	1 × 10^18^

**Table 2:** Parameters for the defects in materials and at interfaces.

Parameters	TiO_2_	CH_3_NH_3_SnI_3_	Cu_2_O	TiO_2_/CH_3_NH_3_SnI_3_ interface	CH_3_NH_3_SnI_3_/Cu_2_O interface
Defect type	Neutral	Neutral	Neutral	Neutral	Neutral
*σ*_*n*_ (cm^−2^)	1 × 10^–15^	2.5 × 10^–13^	1 × 10^–15^	1 × 10^–15^	1 × 10^–15^
*σ*_*p*_ (cm^−2^)	1 × 10^–15^	8.5 × 10^–15^	1 × 10^–15^	1 × 10^–15^	1 × 10^–15^
Energy distribution	Single	Gaussian	Single	Single	Single
Energy level with respect to *E*_*v*_ (above *E*_*v*_) (eV)	0.600	0.650	0.100	0.600	0.600
Characteristic energy (eV)	–	0.100	–	–	–
*N*_*t*_ (cm^−3^)	1 × 10^14^	3.029 × 10^16^ (variable)	1 × 10^14^	1 × 10^10^	1 × 10^10^

## Results and discussion

**Figure 2:**
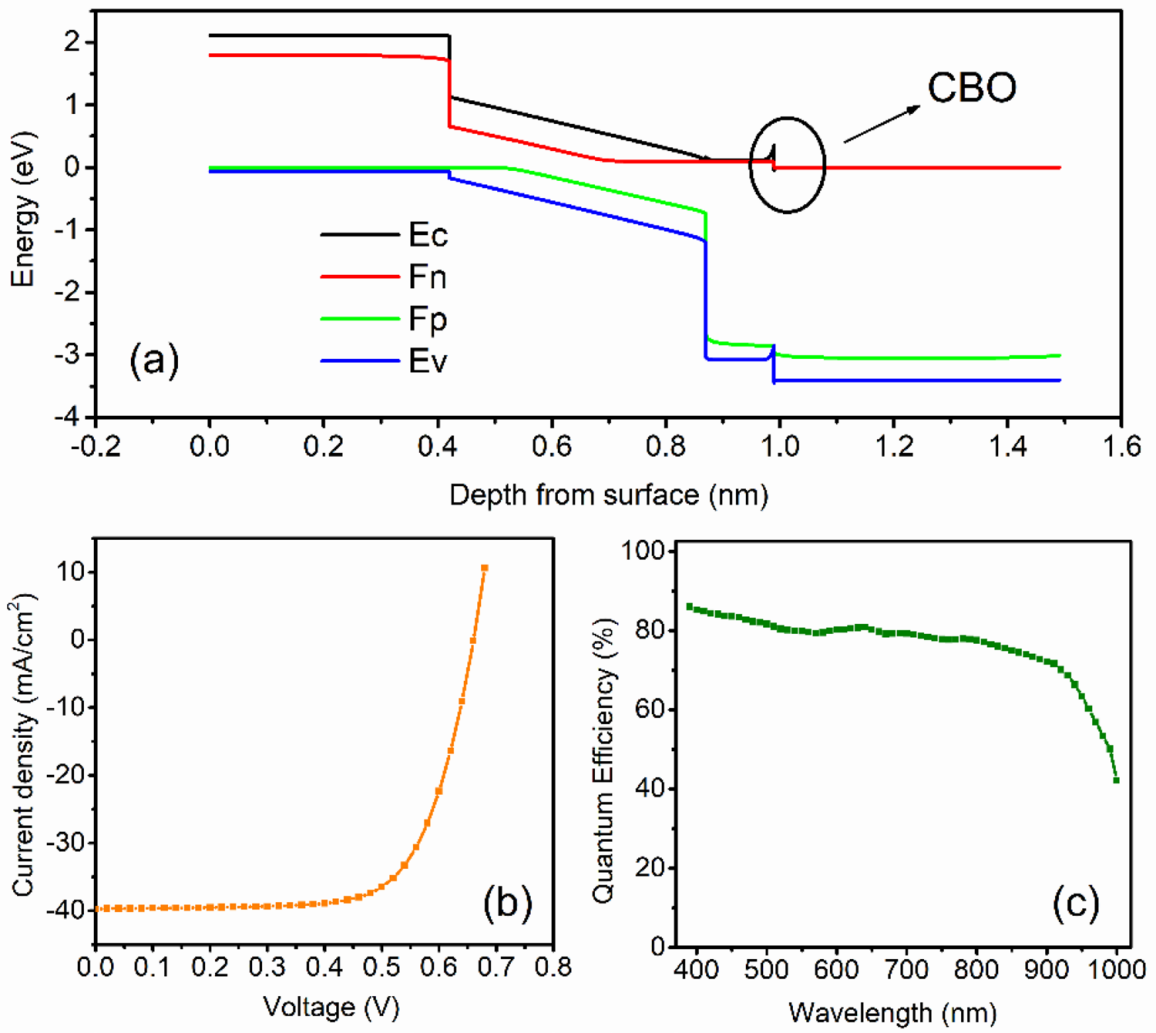
(**a**) Energy band diagram, (**b**) J_sc_ and (**c**) quantum efficiency of PSC.

With these initial parameters in Tables 1 and 2, energy band diagram, *J-V* characteristic and quantum efficiency of the cell was plotted, as shown in Fig. 2a–c, respectively. After illumination, electron–hole pairs are generated inside the absorber layer. Due to the junction field electrons and holes move towards ETL and HTL, respectively. These electrons and holes are collected at the cathode and anode, respectively and generates a voltage. The *J*_*sc*_ of 39.72 mA/cm^2^, *V*_*oc*_ of 0.66 V, *FF* of 69.82%, and *PCE* of 18.31% are obtained. The *J*_*sc*_ of the device depends upon the absorption coefficient, thickness and mobility of the active material. The higher the absorption coefficient, the higher the photo current will be^9,12,29,39,43^. The second important parameter is the thickness of the absorber. It must be thick enough to absorb the highest cut off wavelength of the incident solar radiation^27,29^. Apart from that, mobility plays a very crucial role for getting the high *J*_*sc*_. Ideally, the *J*_*sc*_ is equivalent to solar cell current after illumination. Ma et al. and Stoumpos et al. reported the very high value of mobility of electron (2000 cm^2^/Vs) and hole (300 cm^2^/Vs) for CH_3_NH_3_SnI_3_ sample synthesized by open tube method^28,44^. Similar values of carriers mobilities have been utilized by Lazemi et al. and stated a high value of *J*_*sc*_ (~ 37 mA/cm^2^)^27^. In this simulation, the mobility of electron and hole was adopted from recently studied researcher^27,28,43,44^. Since, current density is linearly proportional to the mobility of charge carriers, and hence the high value of *J*_*sc*_ was achieved. However, Devi et al.^45^ and Khattak et al.^46^ have considered the significantly smaller and identical values of electron and hole mobilities, which are 1.6 cm^2^/Vs and 0.16 cm^2^/Vs, respectively and reported good *J*_*sc*_ (~ 30 mA/cm^2^). Another aspect is that diffusion length is proportional to the square root of mobilit^45^. Hence, diffusion length becomes more for high mobility of charge carrier, and hence recombination of charge carriers decreases. This may be other reasons for getting the comparatively higher value of *J*_*sc*_ as compare to recently reported results^45,46^. The simulated device performance is auspicious and consistent with the tin-based PSC^27,29,39^. The quantum efficiency curve covers the entire visible spectrum, which is in good accordance with the recently published results^47,48^. Further enhancement in photovoltaic performance is possible. Figure 2a displays the energy band diagram of PSC. The positive conduction band offset (CBO) of about 0.36 eV is observed at absorber/ETL interface. Due to this positive CBO, a spike is formed at the absorber/ETL interface. This spike acts as a barrier for photo-generated electron flow towards the electrode^49–52^.

### Absorber thickness

**Figure 3:**
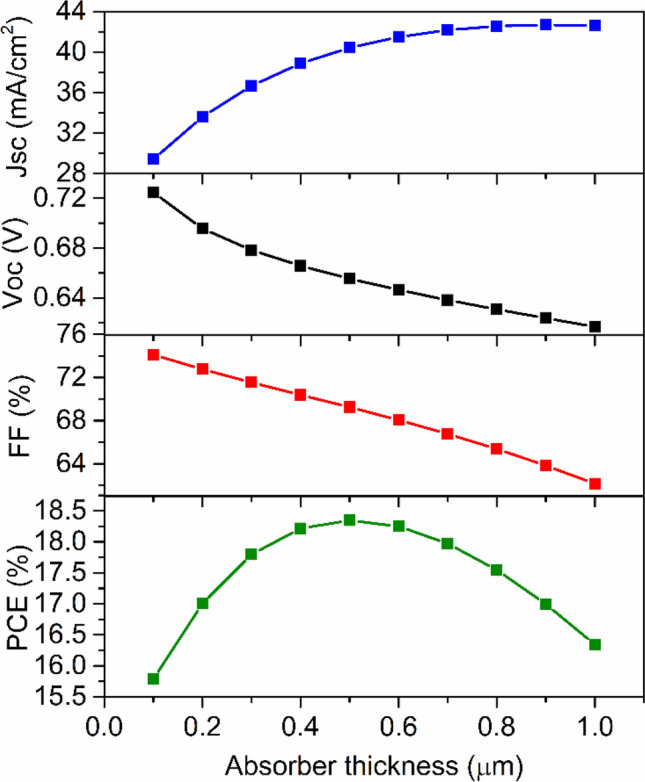
Photovoltaic response as a function of absorber layer thickness.

The absorber layer plays a significant role in the performance of device. The previously published report shows that the photovoltaic parameters such as *J*_*sc*_, *V*_*oc*_, *FF* and *PCE* are influenced by the absorber layer thickness^27,39^. To get the absorber layer's role in the device simulation, the absorber layer's thickness was varied from 100 to 1000 nm, and other parameters tabulated in a Tables 1 and 2 remain the same. The simulation results, i.e., the variation of photovoltaic parameters concerning the absorber layer's thickness, is shown in Fig. 3. It is observed that *J*_*sc*_ increases steeply up to 700 nm and then varies slowly with thickness. The large value of *J*_*sc*_ was obtained (~ 42.70 mA/cm^2^) with a thickness 900 nm is mainly due to the large absorption coefficient of the perovskite^29^. *V*_*oc*_ falls off smoothly, which may be attributed to the enhanced recombination of free charge carriers in the thicker absorber^27^. The decreasing value of *FF* with respect to absorber thickness may be due to the increased series resistance^29,53^. In addition, *PCE* initially increases and reaches a maximum (~ 18.36%) at 500 nm and decreases with a further increase in absorber thickness. Firstly, the absorber thickness is smaller than the diffusion length of charge carriers; therefore, most of the charge carriers reach at the electrodes, and therefore *PCE* increases. However, recombination occurs for thick absorber layer, and hence *PCE* decreases with a further increase in thickness^27,29,39^.

### Acceptor carrier concentration (N_A_) of the absorber

**Figure 4:**
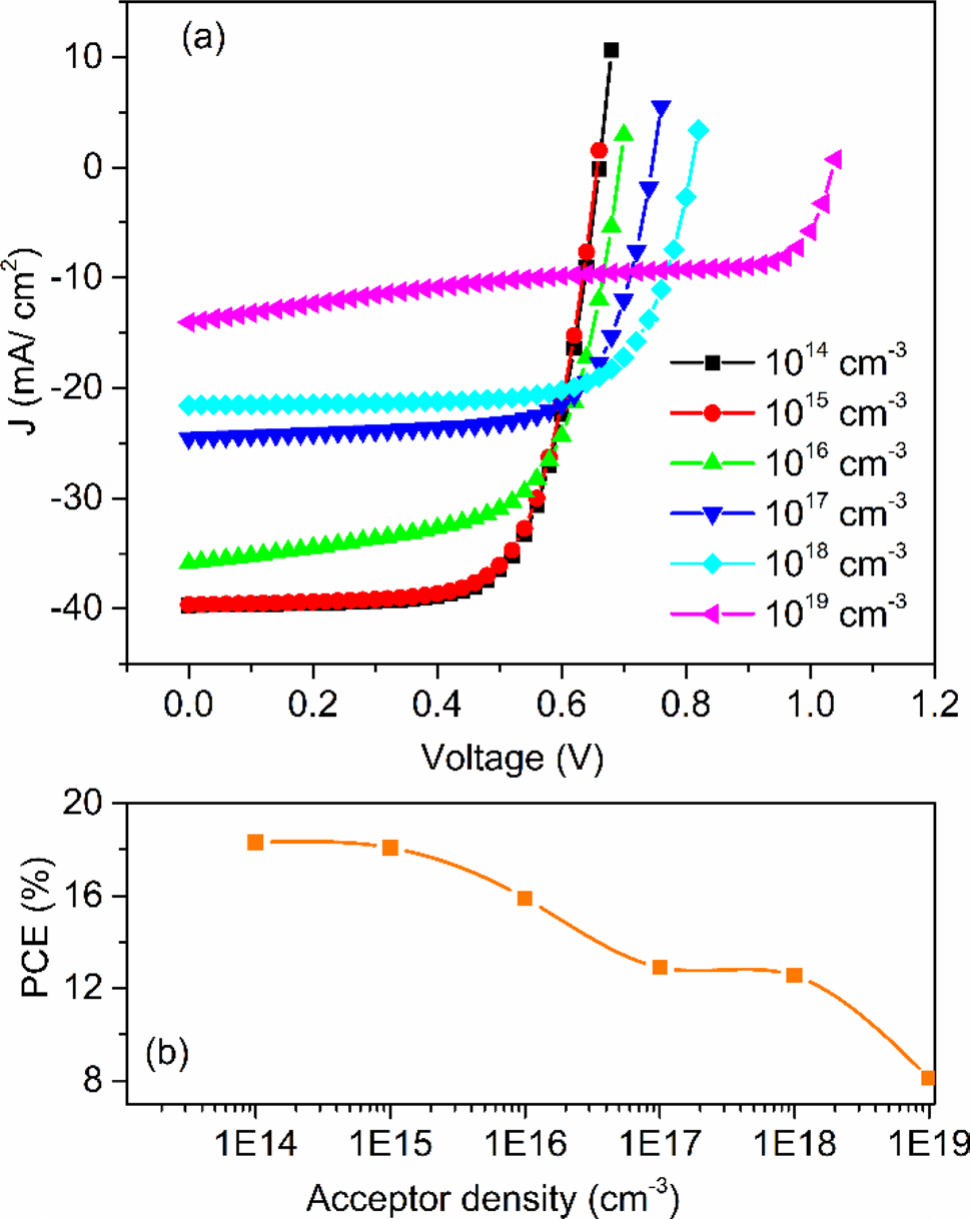
(**a**) *J-V* curve for different acceptor density and (**b**) *PCE* versus acceptor density.

Apart from the absorber layer thickness, the photovoltaic cell's device performance is significantly affected by the acceptor density of holes in the absorber layer. CH_3_NH_3_SnI_3_ oxides in which Sn^2+^ is converted into Sn^4+^ (self-doping process) when the device is exposed to air. Unfortunately, this process deteriorates the performance of the device and making it a *p*-type semiconductor^28^. Addition of SnO_2_ suppresses the formation of Sn^2+^ to Sn^4+^^15,43,54^. Feng et al. calculated the dark carrier density of 10^14^ cm^−3^ to 10^17^ cm^−3^ by Hall- effect measurement^55^. Takashi et el. found out that the hole concentration in the CH_3_NH_3_SnI_3_ absorber layer can be varied up to 10^19^ cm^−3^^56^. Therefore, to get to know how acceptor doping concentration affects the photovoltaic parameters, the acceptor density of the CH_3_NH_3_SnI_3_ layer was varied from 10^14^ to 10^18^ cm^−3^. Figure 4 provides the variation of *J-V* characteristics and *PCE* with respect to acceptor densities of the perovskite layer. The slight change in photovoltaic parameters up to 10^15^ cm^−3^ acceptor concentration implies that the generation rate of photo-generated carriers does not change with acceptor densities under the incident of the same photon number^39^. With increasing the acceptor doping concentration, the Fermi energy level of the hole decreases and hence *V*_*oc*_ increases, as shown in Fig. 4a. Another aspect is that built-in potential increases with increasing the acceptor doping concentration. Due to this, charge separation promotes and hence *V*_*oc*_ increased. However, initially, *J*_*sc*_ decreases slightly up to 10^15^ cm^−3^ and then decreases drastically. It may be due to the increase in the recombination rate of charge carriers inside the perovskite absorber layer^39^. However, *PCE* drops rapidly when *N*_*A*_ exceeds 10^15^ cm^−3^. The absorber layer's defect state leads to a considerable drop in power conversion efficiency, as exposed in Fig. 4b.

### Defect density (N_t_)

**Figure 5:**
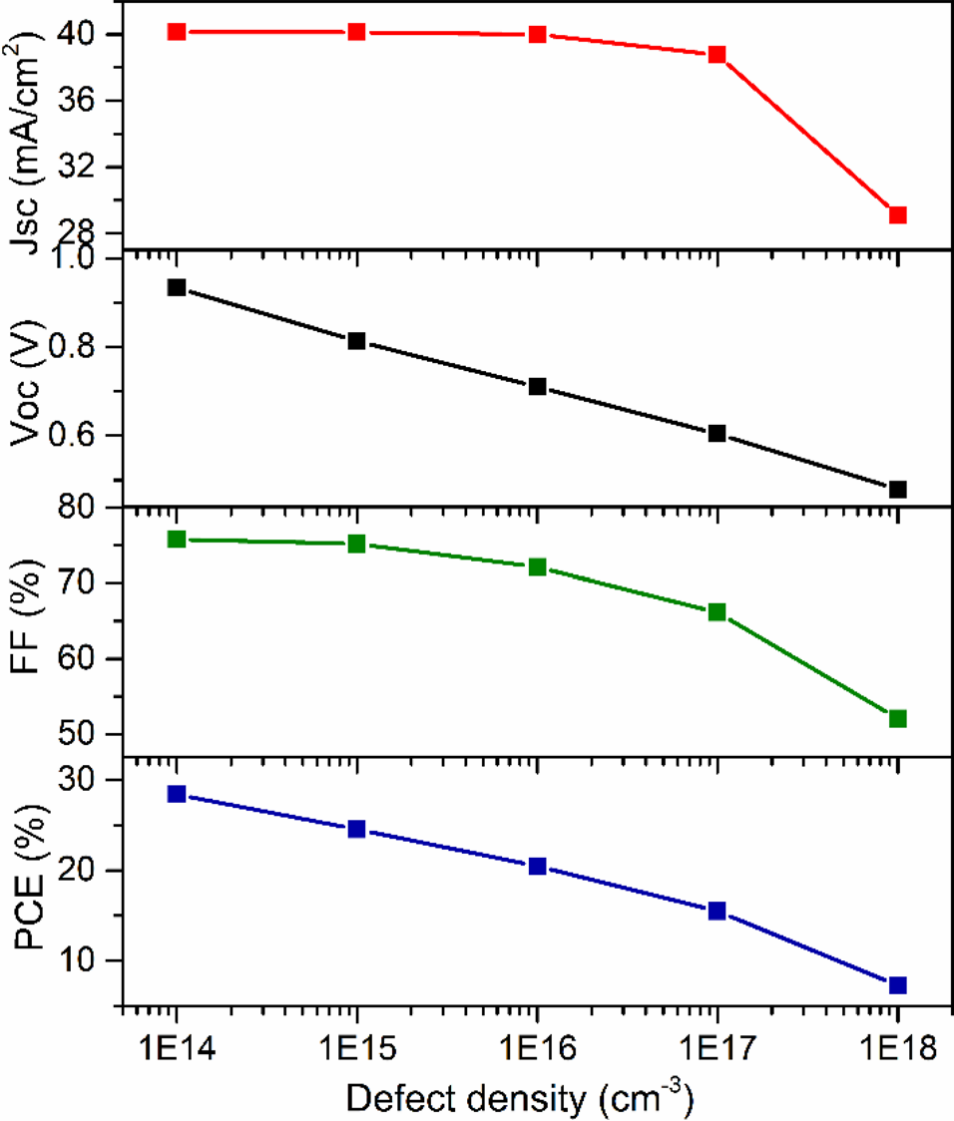
Photovoltaic response as a function of defect density.

The effect of defect density of absorber was also investigated. Defects are inevitable in absorber layer. They exist in the bulk and at surfaces. In perovskite absorber layer, defects present in the form of point defects such as lattice vacancy, interstitial, Schottky and Frenkel defects. Apart from that, the higher order defects like dislocations and grain boundaries may also be present^57^. The self-doping process, which makes the semiconductor *p*-type, produces impurity defect in absorber layer^15,28,43,55^. These defects introduce deep or shallow levels in the energy band gap^57^. As a result of these defects, charge carriers can trap and facilitate non-radiative electron–hole recombination^27,39^. It is noted that the diffusion length of charge carriers is increased up to ~ 3 µm in Sn-based perovskite absorber layer using tin-reduced precursor solution^58^. Since, diffusion length of charge carriers is related to the defect density^45^. Therefore, in order to see the effect of diffusion length on photovoltaic responses, diffusion length of electron was varied from 0.046 to 4.6 µm by changing defect density from 10^18^ to 10^14^ cm^−3^^59^. Similar change in defect density has also been adopted by Lazemi et al., Du et al. and Hao et al.^27,30,39^. Based on these studies, the defect density was varied from 10^14^ to 10^18^ cm^−3^ and depicted its variation on photovoltaic properties in PSC, as shown in Fig. 5. It is observed that the performance of the device improved with the reduction of defect density. The absorber layer's initial defect density was set to be 3.029 × 10^16^ cm^−3^ (as per Table 2). Because for this value of defect density, the diffusion length of electron and hole is nearly similar to experimentally observed values^28^. When the defect density is 10^15^ per cm^−3^, the cell performance is significantly improved, attaining the *J*_*sc*_ of 40.13 mA/cm^2^, *V*_*oc*_ of 0.81 V, *FF* of 75.17% and *PCE* of 24.54%. Now, further decrease of *N*_*t*_, from 10^15^ to 10^14^ cm^−3^, slight variation is observed in *J*_*sc*_ (40.14 mA/cm^2^) and *FF* (75.78%) but considerable changes occurred in *V*_*oc*_ (0.93 V) and *PCE* (28.39%). However, experimentally, it is not easy to synthesize a material with such a low value of defect density^39^.

**Figure 6:**
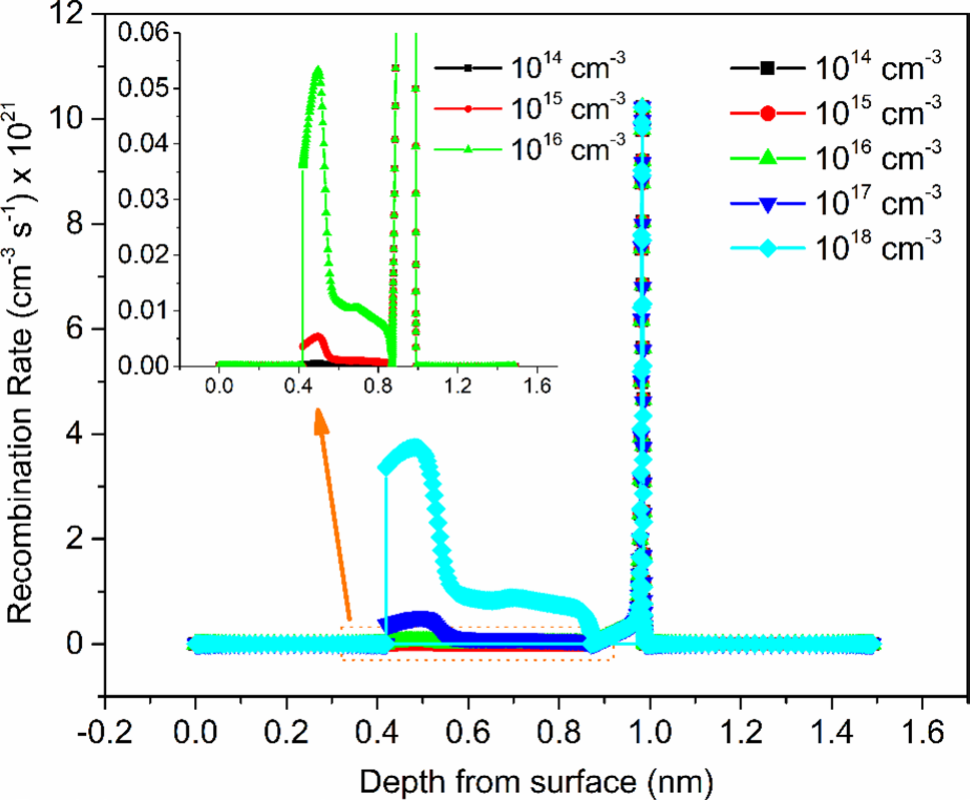
Variation of recombination rate with depth from surface for various defect density along with inset shows the close-up image.

The Shockley–Read–hall (SRH) recombination model can be utilized to get information about the influence of the absorber layer's defect density on device performance^27,29,52^. To get the influence of *N*_*t*_ on the performance of the device acutely, the effect of defect density on the recombination rate based on the SRH recombination model was studied. Figure 6 shows the variation of recombination rate with depth from the surface for different value of *N*_*t*_. It is detected that with increasing the defect density recombination rate increases, which is the reason for the reduction of cell performance with the increased value of defect density. Since, recombination rate increases with increasing the defect density; therefore, *V*_*oc*_ decreases with increasing the defect concentration, as shown in Fig. 5.

According to SRH model, the recombination rate (R) can be expressed like

4 $$R= \frac{{\tau }_{n,p }^{-1}(np-{n}_{i}^{2})}{n+p+2{n}_{i}coh\left(\frac{{E}_{t}-{E}_{i}}{kT}\right)}$$

where, *τ*_*n,p*_, *n*, *p*, *n*_*i*_, *E*_*i*_ and *E*_*t*_ are the lifetime of charge carriers, the density of electron, the density of hole, intrinsic density, intrinsic energy level and energy level of the trap defects, respectively.

Lifetime of charge carriers is given by

5 $${\tau }_{n,p}= \frac{1}{{\sigma }_{n,p}{v}_{th}{N}_{t}}$$

where, *σ*_*n,p*_, *v*_*th*_ and *N*_*t*_ are the capture cross section of charge carriers, velocity of charge carriers, and the absorber layer's defect density, respectively. Therefore, with increasing the defect density, the relaxation time of charge carriers decreases (as per Eq. 5) and hence recombination rate increases (according to Eq. 4) as confirmed by Fig. 6.

The interface recombination depends upon the conduction band offset between the buffer and absorber layer. The interface recombination at the absorber/buffer interface reduces due to the creation of positive CBO^49–51^. Minemoto et al. theoretically studied the effect of CBO at the absorber/buffer interface^49^. He reported that about 0.3 eV CBO offset minimizes the recombination at the interface due to this photovoltaic parameter increases. Hence, the recombination rate is significantly low at the absorber/ETL interface as compared to previously reported results^39,52^.

The diffusion length of charge carriers can be written like

6 $${L}_{D}=\sqrt{D\tau }$$

where, *D* is the diffusion coefficient. Since, the diffusion coefficient is proportional to the mobility of charge carriers and the mobility of electron (~ 2000 cm^2^/Vs) and hole (300 cm^2^/Vs) is large as experimentally observed by various researchers^30,44^. Due to the large value of mobility, diffusion length is large, which is why obtaining the very high value of *PCE* (28.39%). Because of the low recombination rate and large diffusion length, a very high value of *PCE* was achieved. Hence, the obtained outcomes are found to be better than previously published results^27,29,39,60^.

### Metal electrode work function

**Figure 7:**
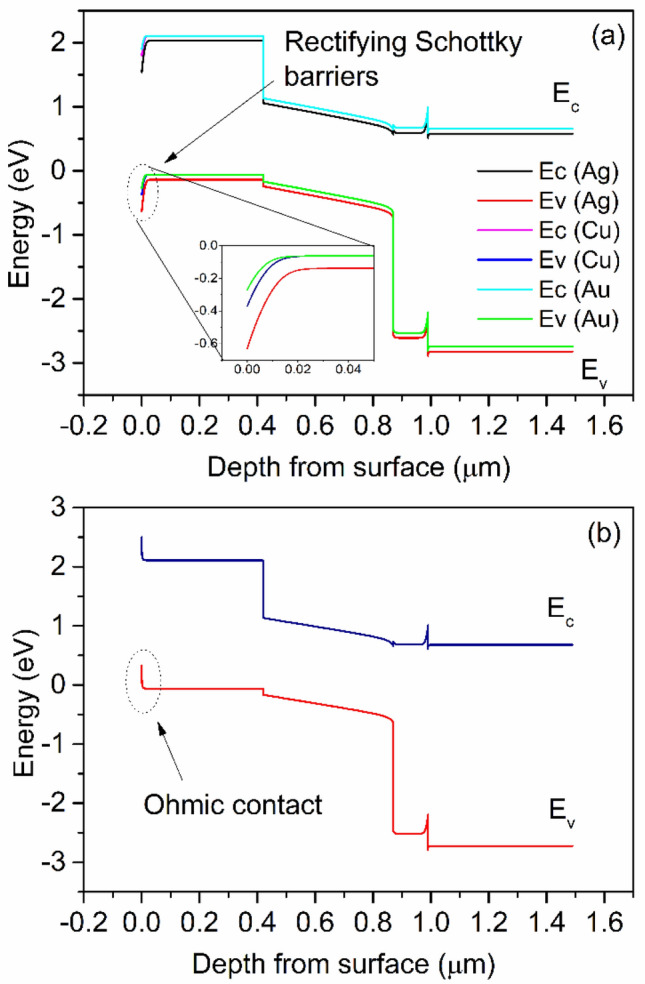
Band diagram of PSC with various anode materials (**a**) Ag, Cu, Au and (**b**) Pt.

**Figure 8:**
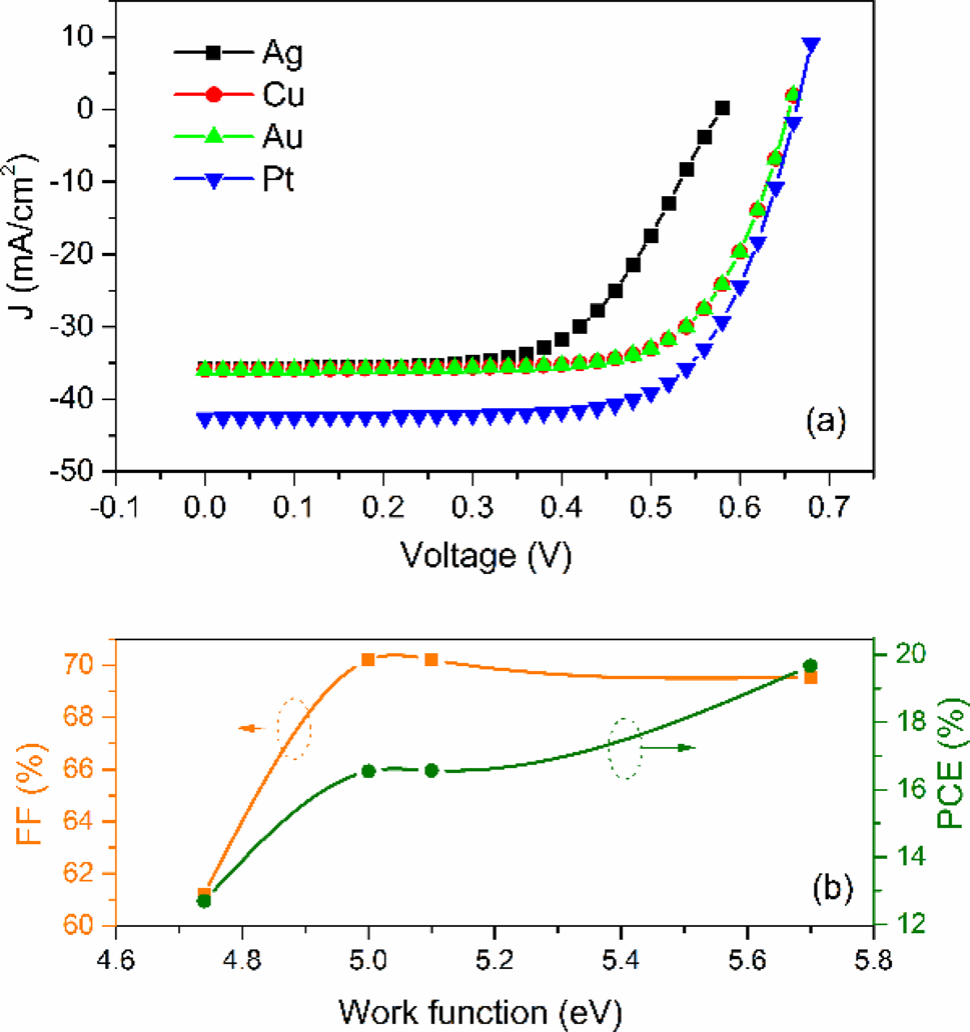
(**a**) *J-V* characteristics with varied work function (**b**) *FF* and *PCE* as a function of work function of anode materials.

To study the ohmic or rectifying behaviour at metal contact/HTL interface, a work function study was carried out by varying various anode materials. Simulation was done using Ag, Cu, Au and Pt as an anode for PSC. The work function of Ag, Cu, Au and Pt are 4.74 eV, 5.0 eV, 5.1 eV and 5.7 eV, respectively^61,62^. The energy band diagram with various anode materials is shown in Fig. 7a,b. As clearly shown that the barrier layer for hole increases with decreasing the wave function of contact materials (Fig. 7a). Figure 8a,b presents the anode material's effect on *J-V* characteristics and photovoltaic properties of PSC. We can see that *PCE* decreases with decreasing the work function of the anode. In the case of Ag, Cu and Au, the anode's work function is less than the work function of Cu_2_O^61,62^. Hence, a rectifying Schottky barrier contact was formed for Ag, Cu and Au anode materials at an anode/Cu_2_O interface, as indicated by the dashed oval frame in Fig. 7a. This Schottky barrier hinders the hole transport to the anode, decreasing the *FF* and *PCE* as confirmed by Fig. 8b^29^. However, in the case of Pt anode, the work function of Pt is higher than the work function of Cu_2_O^63^. The ohmic contact was formed at an anode/Cu_2_O interface, as indicated by the dashed oval frame in Fig. 7b. The ohmic contact allows the hole transport at the interface^62^. Therefore, further improvement in *J*_*sc*_ (42.63 mA/cm^2^) and *PCE* (19.67%) are observed as shown in Fig. 8.

The surface potential energy barrier (${\phi }_{B}$) at the anode/Cu_2_O interfaces is given by

7 $${\phi }_{B}=\frac{{E}_{g}}{q}+\chi -{\phi }_{M}$$

Here, *E*_*g*_ is the band gap of Cu_2_O, $\chi$ is the electron affinity of Cu_2_O and ${\phi }_{M}$ is the anode's work function. Due to the decrease in the value of work function the surface potential energy barrier increases (as per Eq. 7), hence the *FF* and *PCE* decreases.

## Conclusion

Lead-free CH_3_NH_3_SnI_3_ perovskite as light harvester is investigated. A planner heterojunction perovskite solar cell with the structure FTO/TiO_2_/CH_3_NH_3_SnI_3_/Cu_2_O/anode was numerically analysed. Photovoltaic parameters were optimized with respect to several factors such as absorber layer thickness, acceptor density, defect density and work function of anode materials. The optimized perovskite thickness of 500 nm significantly enhances the *PCE* (18.36%). Reducing the defect density and improving the Sn^2+^ stability of absorber layers are the critical issues for future research, which might be resolved by refining the device's fabrication techniques. The results indicated that the appropriate defect density improves the cell performance; however excessive concentration leads to a higher recombination rate of charge carriers and poor cell performance. The Schottky junction was formed at an anode/Cu_2_O interface for lower work function contact materials; therefore, high work function material is necessary for ohmic contact like Pt. The reported CH_3_NH_3_SnI_3_-based PSC provide a viable path to realizing environmentally benign, low-cost, and high-efficiency PSC.

## Data Availability

The data that support the findings of this study are available from the corresponding author upon reasonable request.
